# Leriche syndrome with wound myiasis

**DOI:** 10.11604/pamj.2024.48.155.43420

**Published:** 2024-08-06

**Authors:** Yoen Young Chuah, Yu-Ren Li

**Affiliations:** 1 Department of Internal Medicine, Ping Tung Christian Hospital, Division of Gastroenterology and Hepatology, Pingtung, Taiwan,; 2 Department of Nursing, Meiho University, Pingtung, Taiwan,; 3 Department of Internal Medicine, Ping Tung Christian Hospital, Division of Intensive Care Unit, Ping Tung, Taiwan

**Keywords:** Leriche syndrome, myiasis, gangrenous wound

## Image in medicine

A 67-year-old man presented with progressive shortness of breath and claudication for one week. He has a thirty-year history of heavy smoking. Physical examination revealed a gangrenous, suppurative wound and myiasis on the right great toe (A, B). In addition, a non-pulsating right popliteal artery was observed. His laboratory analysis revealed leukocytosis, bandemia (an increased number of immature white blood cells), and an elevated C-reactive protein (CRP) level. A computed tomography scan uncovered atherosclerosis affecting the distal abdominal aorta, iliac arteries, and femoral vessels that is compatible with Leriche syndrome (C). Antibiotic treatment with cefoperazone/sulbactam was administered, along with surgical debridement of the gangrenous toe (D). To improve local blood circulation, percutaneous transluminal angioplasty (PTA) was performed on the right common femoral artery (E, F). Dual antiplatelet agents were prescribed, and the patient was advised to quit smoking. Our patient has had an uneventful clinical course and was successfully discharged after a three-week hospitalization. Leriche syndrome is a progressive disease characterized by claudication, erectile dysfunction, and diminished distal pulses. Hyperlipidemia, diabetes mellitus, and smoking are the risk factors. Long-term smoking history in our patient is a prominent risk factor attributable to Leriche syndrome, which causes hypoperfusion at distal extremities that may lead to wound formation. Moist, gangrenous wounds associated with Leriche syndrome may attract flies, increasing the risk of myiasis. This presentation represents the initial documented case of wound myiasis in a patient diagnosed with Leriche syndrome, thus offering valuable insights into this infrequently encountered clinical association.

**Figure 1:**
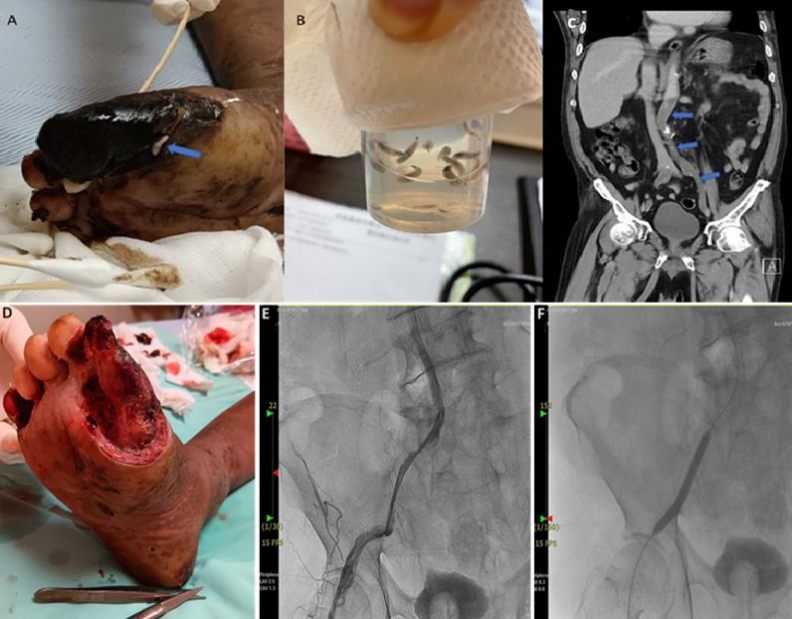
A) the arrow indicating a gangrenous right big toe with visible maggot infestation; B) maggots retrieved from the gangrenous wound of the right big toe; C) a non-enhancement of the distal abdominal aorta extending to the femoral arteries (left), marked by arrows, which is compatible with Leriche syndrome; D) the gangrenous right big toe has been surgically debrided to promote better wound healing; E) stenosis and angioplasty of right common femoral artery; F) angioplasty intervention with balloon dilatation over the stenotic right common femoral artery

